# Vehicle Load Estimation Using the Reaction Force of a Vertical Displacement Sensor Based on Fiber Bragg Grating

**DOI:** 10.3390/s22041572

**Published:** 2022-02-17

**Authors:** Sung-Wan Kim, Da-Woon Yun, Dong-Uk Park, Sung-Jin Chang, Jae-Bong Park

**Affiliations:** 1 Seismic Research and Test Center, Pusan National University, Yangsan 50612, Korea; swkim09@pusan.ac.kr (S.-W.K.); ardw818@pusan.ac.kr (D.-W.Y.); kwenry@pusan.ac.kr (D.-U.P.); 2 Management Office, Korea Authority of Land & Infrastructure Safety, Jinju 52856, Korea; jbpark@kalis.or.kr

**Keywords:** bridge bearing, fiber Bragg grating, vertical displacement sensor, reaction force, vehicle load

## Abstract

Accurately calculating the vehicle load acting on a bridge at any one time is crucial to determining the integrity and safety of the bridge. To ensure this integrity and safety, information on the types, characteristics, and load of vehicles that regularly cross the bridge is very important in terms of its structural adequacy and maintenance. In this study, the vehicle load that a bridge will be subjected to was estimated using the reaction force response at the support. To estimate this response to the reaction force, a vertical displacement sensor, developed based on Fiber Bragg Grating (FBG), was applied to the Eradi Quake System (EQS), a commercially available bridge bearing. This vertical displacement sensor can measure the vertical load and has the advantage of being easy to attach and detach. To verify the performance and accuracy of this sensor, this study conducted numerical analysis and vehicle loading tests. It found that the vehicle load can be estimated from the reaction force response, as measured by the vertical displacement sensor on the bridge.

## 1. Introduction

With the growing interest in bridge maintenance technology, the number of bridges that require a structural safety assessment due to performance degradation and aging has rapidly increased [1,2]. Bridges currently in operation are subjected to performance degradation due to the applied load, deterioration of components, and changes in the external environment [3,4]. It is essential for bridge maintenance to continuously examine such changes in the condition of the bridge and to evaluate bridge safety by continually monitoring them [5,6,7,8,9]. Therefore, there is an urgent need to develop technologies to secure bridge safety in relation to the growing number of old bridges worldwide [10,11,12].

To identify changes in the condition of a bridge, it is important to accurately calculate the total loads acting on it [13]. Important loads acting on a bridge include dead loads and live loads (e.g., vehicle loads, wind loads, and seismic loads). Since there is no significant change in dead loads after construction, it is expected that damage to the bridge will primarily be due to the characteristics and dynamics of its live loads. Thus, it is important to identify the live loads of a bridge when examining its safety [14]. Among the load factors that may occur on a highway bridge over the long term, vehicle load is the most important factor, in addition to the temperature load and concrete creep. A bridge can, therefore, only be efficiently maintained if the vehicle loads it is subjected to are clearly identified [15,16,17].

Representative methods to measure the vehicle load acting on a bridge are the Weigh-In-Motion (WIM) system [18] and Bridge Weigh-In-Motion (B-WIM) system [19]. The WIM system measures the axial load and total load of a vehicle by installing axle load sensors on the pavement. This system significantly affects the vehicle load measurement results due to the dynamic interaction between the vehicles and the road surface, and may cause signal distortion due to pavement deformation and damage. In addition to high installation and maintenance costs, the WIM system also requires the vehicles to travel at a low speed for accurate vehicle load measurement. To address these problems, various WIM systems were developed [20,21,22] along with methods using such WIM systems to analyze the vehicle load [23]. A WIM system that performs automatic correction was developed [24] to improve the accuracy of vehicle load measurement, while a study was also conducted to detect bridge damage using a WIM system [25].

Unlike the WIM system that directly measures the vehicle load, the B-WIM system measures the vehicle load using the bridge itself as a scale. Studies have been conducted to measure the vehicle load acting on a bridge using the B-WIM system. The B-WIM system was developed to estimate the vehicle load by applying strain sensors and axle detectors [19], and a portable B-WIM system that uses the acceleration response was also proposed to perform this estimate [26]. In addition, a B-WIM system that uses the shear force response of a bridge was developed [27,28] along with a method to estimate the vehicle load in the time domain using the equation of motion [29]. A study was conducted to analyze the influence of the heavy vehicles’ travel characteristics using the B-WIM system [30] as well as the influence of live and fatigue loads on highway bridges, also using the B-WIM system [31]. There was also a study that monitored the influence line of a short-span bridge using the B-WIM system [32], and another study was conducted on methods to improve the accuracy of the B-WIM system [33,34,35,36,37].

Since the B-WIM system was first proposed by F. Moses [19], most B-WIM systems have required the attachment of axle detectors to the road surface and strain sensors to the bottom of the bridge superstructure. The axle detectors measure the speed, axial distance, and position of a vehicle. Using the strain sensors, the axial load and total load of a vehicle are then calculated through the simultaneous input of the strain on a bridge member, as measured using the axle detectors. Using this method, however, it is difficult to install and maintain axle detectors because they have to be located on the road surface itself. Consequently, without installing axle detectors, studies had to be conducted to estimate the speed, axial distance, axial load, and total load of a vehicle using only the strain sensors installed at the bottom of the bridge superstructure [38,39,40,41].

When measuring strain response using an electrical resistance-type strain gauge, there is a disadvantage of electrical noise being increasingly generated as the cable connecting the sensor and data acquisition system becomes longer. In contrast, the measurement of strain response using the Fiber Bragg Grating (FBG) sensor is not affected by electrical noise and makes multiple measurements possible. In addition to requiring a shorter measurement cable, the FBG sensor is simpler to install and remains stable in harsh environments characterized by a lot of humidity and dust. The FBG sensor was, therefore, used in this study for the same reason that it is preferred for use when measuring the strain response for various infrastructures, such as roads and bridges [42,43,44].

In this study, a vertical displacement sensor was developed based on an FBG that can measure the vehicle load using the reaction force response at the support without having to install sensors on the bridge road surface itself. This FBG was designed precisely to address the problems that were described in the preceding discussion. The fact that the FBG can be easily attached and detached is a great advantage as the measurement of the reaction force response typically entails the complicated process of replacing the bridge bearings when the sensors malfunction.

Apart from being easy to attach and detach, the vertical displacement sensor developed for this study is reusable. The sensor can be installed on any bridge requiring measurement without replacing the bridge support. This makes it possible to measure the vehicle load passing through the bridge as well as the dead load on the bridge. Therefore, in order to verify the usability of the vertical displacement sensor, a vehicle loading test was performed in a compressive test and on an actual bridge.

## 2. Vertical Displacement Sensor Based on Fiber Bragg Grating

In this study, the vertical displacement sensor, developed based on FBG to measure the vertical load, was applied to the Eradi Quake System (EQS). The EQS is a bridge bearing that performs the same function as a load-carrying bearing to provide resilience between the upper and lower structures, as well as accommodate their rotation. EQS is equipped with a polyurethane disk that allows for the vertical load and rotational displacement of the upper structure at all times. Therefore, it is possible to estimate the vertical load acting on EQS every time the displacement response of the polyurethane disk in the vertical direction is measured.

**Figure 1:**
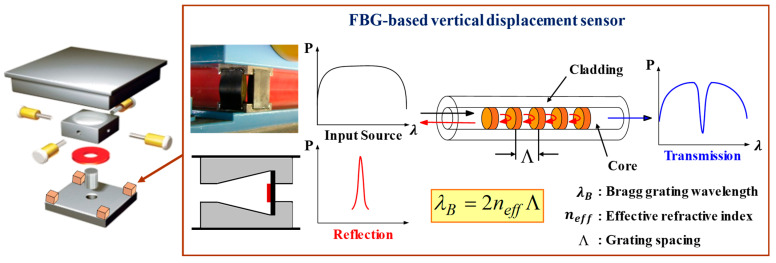
FBG-based vertical displacement sensor applied to EQS.

Figure 1 shows the method of estimating vertical load by applying the vertical displacement sensor to the EQS and measuring the displacement response in the vertical direction. When a broadband spectrum is incident on an optical fiber, the wavelength component that satisfies the conditional expression is reflected by the optical fiber grating. The strain response is measured using the change in wavelength observed through an optical spectrum analyzer. Here, P is the size of the wavelength, λ is the wavelength, $n_{eff}$ is the effective refractive index, and Λ is the grid spacing. In this study, the FBG sensor was fabricated using single-mode fiber 28 (SMF28), which is widely used for general communication and the production of optical fiber sensors. The refraction and period were changed in the grating inscription, and the frequency of the reflected light was changed accordingly [45]. Bragg’s wavelength for the FBG sensor was fabricated with a range from 1514 nm to 1575 nm. Since the polyurethane disc built into EQS accommodates the vertical load acting on the bridge support, the vertical load response is estimated by installing the developed vertical displacement sensors at four corners.

### 2.1. Developed Vertical Displacement Sensor

**Figure 2:**
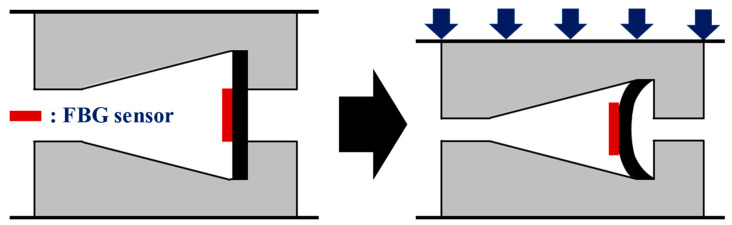
FBG-based vertical displacement sensor.

To measure the vertical displacement of the polyurethane disk, a vertical displacement sensor was developed, as shown in Figure 2. The sensor consists of a beam in the center for measuring the vertical displacement and upper and lower jigs with grooves to fix the beam. In addition, FBG is installed on the beam to measure the strain of the beam. For the vertical displacement sensor developed for this study, the curvature occurs at the center of the beam when vertical displacement is caused by the vertical load. The strain based on the curvature of the beam is measured using FBG, and the vertical displacement response is estimated from the relationship between the strain and that curvature.

If the curvature is assumed to be linear, the same strain occurs at all positions on the surface of the beam because the curvature is the same at the various positions. The relationship between the curvature and the strain can be expressed as Equation (1). Here, r is the radius of curvature of the beam with a length of l, δ is the distance from the neutral axis of the beam cross-section to its thickness, and ε is the strain of the surface.

(1) $$r=\frac{\delta }{\varepsilon }$$

**Figure 3:**
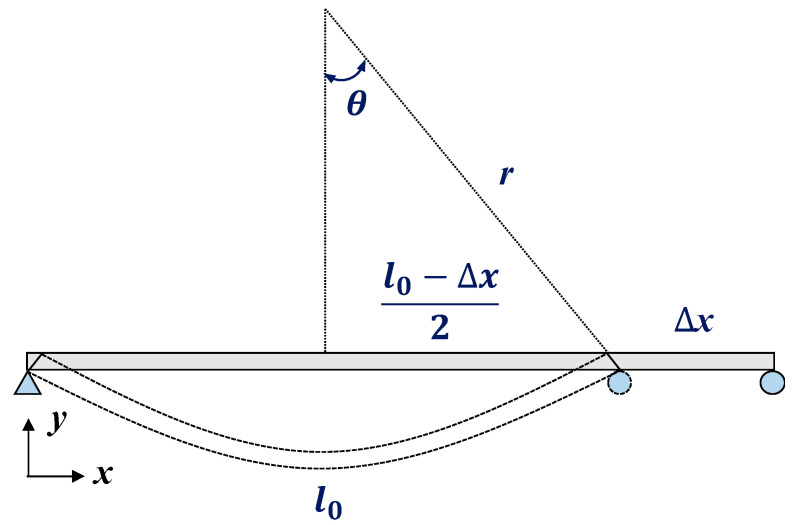
Bending geometry of a simple beam.

When a load is applied on a simple beam, bending occurs with the movement of the roller, as shown in Figure 3. If the beam in Figure 2 is assumed to be a simple beam, the vertical displacement can be estimated. sinθ is obtained from Equation (2) to identify θ. Since rθ=l/2 holds, Equation (3) can be obtained.

(2) $$\sin\theta =\frac{l-\Delta x}{2r}\;$$

(3) $$\sin\left(\frac{l}{2r}\right)=\frac{l-\Delta x}{2r}$$

When Equation (3) is summarized for the vertical displacement (Δx), it can be expressed as Equation (4). The distance from the neutral axis of the beam cross-section to its thickness and the length of the beam are determined over the course of designing the vertical displacement sensor. Therefore, the relationship between the measured strain and the vertical displacement can be obtained as Equation (4). Since Equation (4) diverges if the strain is zero, the sensor was fabricated with vertical deformation to prevent the displacement response from diverging.

(4) $$\Delta x=l-2r\;\sin\left(\frac{l}{2r}\right)=l-2\frac{\delta }{\varepsilon }\;\sin\left(\frac{\varepsilon l}{2\delta }\right)$$

### 2.2. Verification of the Vertical Displacement Sensor Using a Depth Micrometer

**Figure 4:**
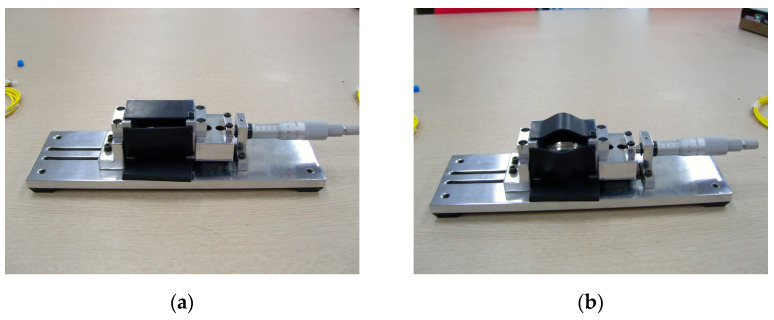
Forced displacement to vertical displacement sensors using the depth micrometer: (**a**) vertical displacement sensors installed in the depth micrometer; (**b**) forced displacement.

**Figure 5:**
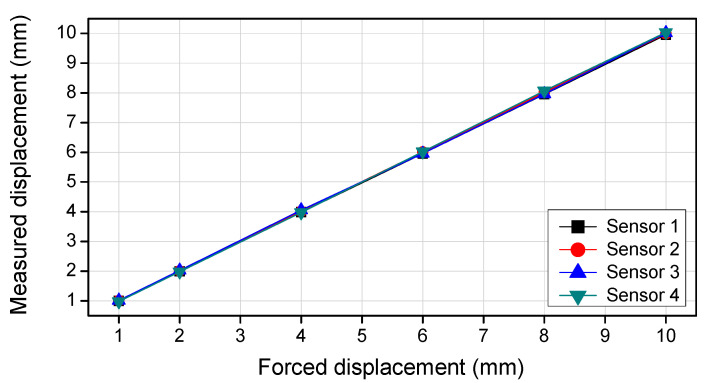
Displacement measurements by vertical displacement sensors.

The specification of the FBG-based vertical displacement sensor is shown in Table 1. In this study, the depth micrometer in Figure 4 was used to check the precision of the developed vertical displacement sensor. The displacement was measured using four vertical displacement sensors, while the forced displacement of the depth micrometer was increased. Figure 5 compares the displacement measurements of the four vertical displacement sensors with the forced displacement of the depth micrometer, and the results are shown in Table 2. The difference in displacement was less than 1%, indicating that the developed vertical displacement sensor has high reliability.

### 2.3. Verification of the Vertical Displacement Sensor Using Compressive Tests

**Figure 6:**
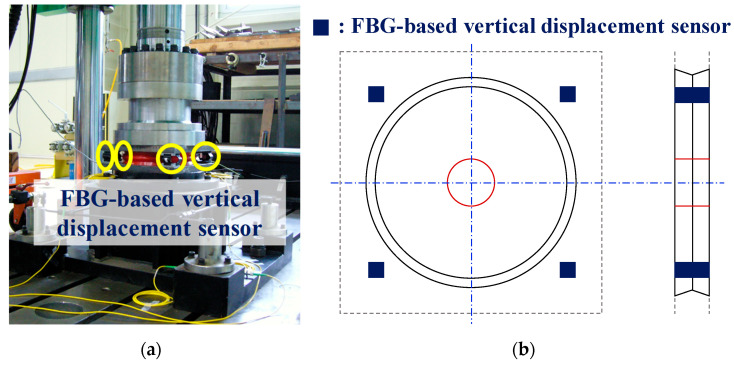
Experimental setup: (**a**) vertical displacement sensors installed in the UTM; (**b**) sensor installation positions.

Compressive tests were conducted to verify the performance of the vertical displacement sensor developed for this study. For the tests, the universal testing machine (UTM) shown in Figure 6a was used. The UTM has a maximum loading capacity of 1000 kN and a maximum stroke of 300 mm. Figure 6b shows the installation position of the sensor. In this study, vertical displacement sensors were installed by installing steel plates at the top and bottom of the polyurethane disk to fully consider their installation conditions on bridges. In addition, four vertical displacement sensors were installed at the edge of the polyurethane disk to prevent eccentricity, as shown in Figure 6b. The average values of the measurements taken by the four sensors were used to estimate the vertical displacement response, taking the eccentricity into account.

**Figure 7:**
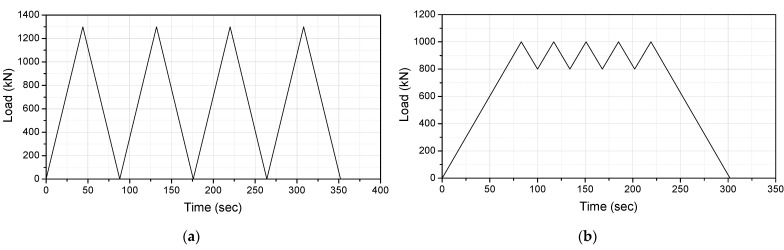
Load conditions in compressive tests: (**a**) Case1; (**b**) Case2.

The compressive tests were conducted under two types of test conditions, as shown in Figure 7. This was to examine whether an accurate displacement response measurement is possible under different loads. In the first experimental condition, applying and removing a load of 1300 kN was repeated, as shown in Figure 7a. In the second experimental condition, a load of ±100 kN was repeatedly applied while a load of 900 kN was, as shown in Figure 7b.

To examine the accuracy and precision of the vertical displacement sensor, an error analysis was conducted using the percent error of Equation (5) and the root mean square (RMS) error of Equation (6). Here, n is the number of measurement data, $\delta_{m}$ is the displacement response measured through the linear variable differential transformer (LVDT) installed in the UTM, and $\delta_{c}$ is the displacement response measured through the vertical displacement sensor.

(5) $$\mathrm{Percent}\;\mathrm{error}=\overset{n}{\underset{i=1}{\sum }}{\left(\delta_{m}-\delta_{c}\right)}^{2}/\overset{n}{\underset{i=1}{\sum }}{\left(\delta_{m}\right)}^{2}$$

(6) $$\mathrm{RMS}\;\mathrm{error}=\sqrt{\overset{n}{\underset{i=1}{\sum }}{\left(\delta_{c}-\delta_{m}\right)}^{2}/n}$$

**Figure 8:**
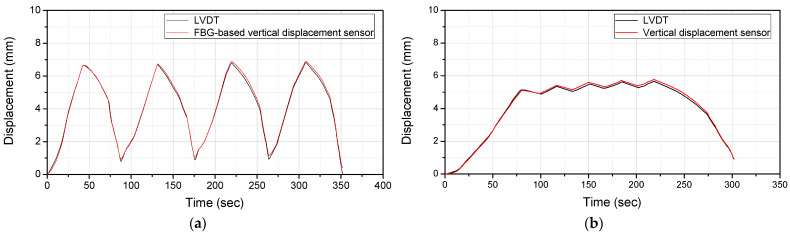
Comparison of the displacement response measured using LVDT and vertical displacement sensor: (**a**) Case1; (**b**) Case2.

Figure 8 compares the displacement response measured through the LVDT installed in the UTM with that measured through the vertical displacement sensor developed for this study. It can be seen that the results are in good agreement with each other. Table 3 shows the results of the error analysis conducted using the percent error and RMS error. The percent error was less than 0.2% and the RMS error was less than 0.5 mm, confirming that the errors were very small. This indicates that a reliable displacement response measurement is possible using the vertical displacement sensor developed for this study.

### 2.4. Verification of the Durability of the Vertical Displacement Sensor Using the Fatigue Test

The primary loads acting on a bridge consist of the dead load attributed to the weight of the bridge and the fatigue load attributed to the vehicles. The vertical displacement sensor developed also accommodates the fatigue load simultaneously with its installation on a bridge. When a vehicle passes through a bridge, deflection occurs in the bridge, and it acts as the fatigue load on the vertical displacement sensor. Therefore, it is necessary to verify the durability of the vertical displacement sensor against the fatigue load.

**Figure 9:**
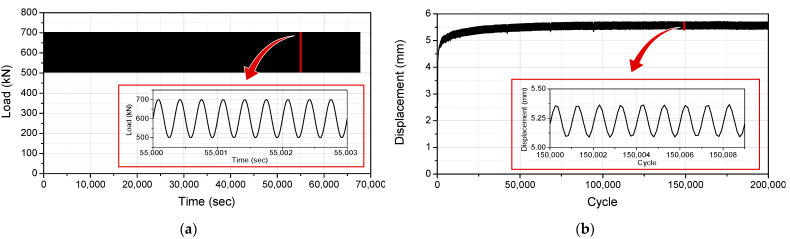
Applied load in the fatigue test and the displacement response measured using the vertical displacement sensor: (**a**) load applied in the fatigue test; (**b**) displacement response measured using the vertical displacement sensor.

The vertical displacement sensor developed for this study is detachable, so it can be replaced when damaged and installed only when required, thus making long-term monitoring possible. Since the sensor is detachable, it will be sufficient to verify its durability against 200,000 cycles of fatigue loading if it is installed only when measurement is required. In the fatigue test, it was assumed that the dead load was 500 kN and a load of 200 kN was repeatedly applied. Thus, loads between 500 and 700 kN were repeatedly applied, as shown in Figure 9a. In addition, the loading frequency was set to 3 Hz.

Figure 9b shows the displacement response measured using the vertical displacement sensor in the fatigue test. The time delay of the polyurethane disk was observed when a constant load was applied, but the vertical displacement response converged to a certain value over time. Table 4 compares the measured displacement according to the number of load cycles. Due to the time delay of the polyurethane disk, the maximum and minimum displacements varied depending on the number of load cycles, but the difference between them was constant at 0.262 mm. This confirmed that the developed vertical displacement sensor can accurately measure the displacement response even after 200,000 cycles of fatigue loading. As the displacement response converges to a constant value over time in the fatigue test, it was found that the dead load of the bridge could be measured. Additionally, since the displacement difference according to the cyclic load has the same value, it was found to be suitable for measuring the vehicle load passing through the bridge.

## 3. Method of Estimating the Vehicle Load Using Reaction Force Response

Heavy vehicles traveling on a bridge act as fatigue loads, which primarily shorten the service life of the bridge. Therefore, bridges can be efficiently maintained by identifying the travel characteristics of passing vehicles.

In this study, the B-WIM system that uses the reaction force response at the support was applied instead of the conventional method of using the strain response measured from the girder of a bridge. Estimation of the vehicle load acting on a bridge using the B-WIM system was performed based on the theory proposed by F. Moses [18].

**Figure 10:**
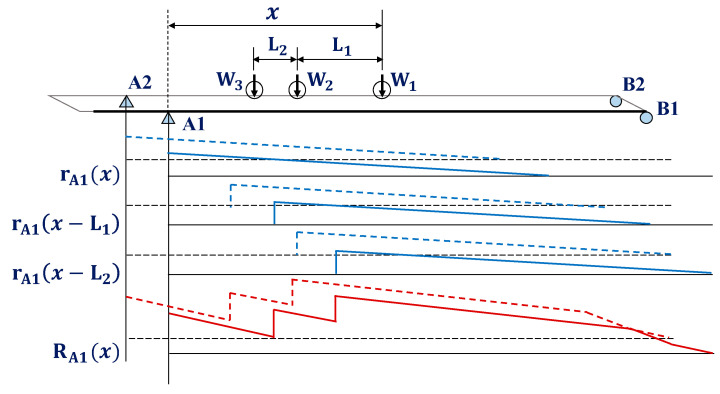
Influence line for the reaction force at the support.

In Figure 10, if the influence line for the reaction force at the support is used, the reaction force response at the support can be expressed as Equation (7). Here, R(x) is the reaction force, $W_{n}$ is the n-th axle weight, and r(x) is the influence line at vehicle position x on the bridge. Since the entry and departure times of the vehicle can be identified using the reaction force response, the speed of the vehicle can be estimated using Equation (8). Here, L is the distance of the support, and ΔT is the time required for the axle to pass the two points.

(7) $$R\left(x\right)=W_{1}r\left(x\right)+W_{2}r\left(x-L_{1}\right)+W_{3}r\left(x-L_{2}\right)\;$$

(8) $$v=\frac{L}{\Delta T}$$

The number of vehicle axles that pass through the bridge is the frequency at which the impact of the measured reaction force response is applied, and the wheel base can be expressed as Equation (9). Here, $l_{AB}$ is the distance between the A axis and B axis of the vehicle, and $\Delta t_{AB}$ is the time difference that the A and B axes pass one point on the bridge.

(9) $$l_{AB}=v\times \Delta t_{AB}\;$$

(10) $$R_{i}\left(t\right)=\overset{NL}{\underset{l=1}{\sum }}\overset{Naxl\left(l\right)}{\underset{k}{\sum }}W_{kl}r_{kli}\left(t\right)$$

Equation (7) can be expressed as Equation (10) if the reaction force at a point is expressed by representing the reaction force by all lanes and girders as a function of time. In Equation (10), $W_{kl}$ is the k-th axle weight in lane l, and $r_{kli}\left(t\right)$ is the influence line value by the k-th axis at time t at the i-th point of line l. NL is the number of lanes, while Naxl(l) is the total number of axles in lane l. The least squares error function is constructed using the static influence lines and measurements at all points, as shown in Equation (11). Here, $E_{i}$ is the least squares error function, $R_{i}\left(t\right)$ is the theoretical reaction force at the i-th point, and $R_{i}{}^{*}\left(t\right)$ is the reaction force measured at the i-th point. Equation (12) is obtained by substituting Equation (10) into Equation (11). Equation (12) can be converted into Equation (13), which can be reconstructed using $W_{n}{}^{*}↔W_{kl}$ and $r_{ni}{}^{*}\left(t\right)↔r_{kli}\left(t\right),$ as shown in Equation (14).

(11) $$E_{i}=\overset{T}{\underset{t=1}{\sum }}{\left[R_{i}\left(t\right)-R_{i}^{*}\left(t\right)\right]}^{2}\;$$

(12) $$E_{i}=\overset{T}{\underset{t=1}{\sum }}{\left[\overset{NL}{\underset{l=1}{\sum }}\overset{Naxl\left(l\right)}{\underset{k}{\sum }}W_{kl}r_{kli}\left(t\right)-R_{i}^{*}\left(t\right)\right]}^{2}$$

(13) $$E=\overset{NG}{\underset{i=1}{\sum }}\overset{T}{\underset{t=1}{\sum }}{\left[\overset{NL}{\underset{l=1}{\sum }}\overset{Naxl\left(l\right)}{\underset{k}{\sum }}W_{kl}r_{kli}\left(t\right)-R_{i}^{*}\left(t\right)\right]}^{2}$$

(14) $$E=\overset{NG}{\underset{i=1}{\sum }}\overset{T}{\underset{t=1}{\sum }}{\left[\overset{NA}{\underset{n=1}{\sum }}W_{n}^{*}r_{ni}^{*}\left(t\right)-R_{i}^{*}\left(t\right)\right]}^{2}$$

Since the sum of the error values must have a minimum value for the axle weight, Equation (14) can be expressed as Equation (15) through partial differentiation with the m-th axle load.

(15) $$\frac{\partial E}{\partial W_{m}}=\overset{NG}{\underset{i=1}{\sum }}\overset{T}{\underset{t=1}{\sum }}\left[\overset{NA}{\underset{n=1}{\sum }}W_{n}^{*}r_{ni}^{*}\left(t\right)\right]r_{mi}^{*}\left(t\right)-\overset{NG}{\underset{i=1}{\sum }}\overset{T}{\underset{t=1}{\sum }}R_{i}^{*}\left(t\right)r_{mi}^{*}\left(t\right)=0$$

If the influence line value is expressed as Equation (16) and the reaction force is expressed as Equation (17), while Equation (15) can be expressed as Equation (18).

(16) $$F_{mn}=\overset{NG}{\underset{i=1}{\sum }}\overset{T}{\underset{t=1}{\sum }}r_{ni}^{*}\left(t\right)r_{mi}^{*}\left(t\right)$$

(17) $$R_{m}=\overset{NG}{\underset{i=1}{\sum }}\overset{T}{\underset{t=1}{\sum }}R_{i}^{*}\left(t\right)r_{mi}^{*}\left(t\right)$$

(18) $$\overset{NA}{\underset{n=1}{\sum }}F_{mn}\times W_{n}^{*}=R_{m}$$

Equation (18) can be expressed as a determinant as shown in Equation (19), which can be summarized for the axle weight {W}, as shown in Equation (20).

(19) [F]{W}={R} 

(20) $$\left\{W\right\}={\left[F\right]}^{-1}\left\{R\right\}$$

**Figure 11:**
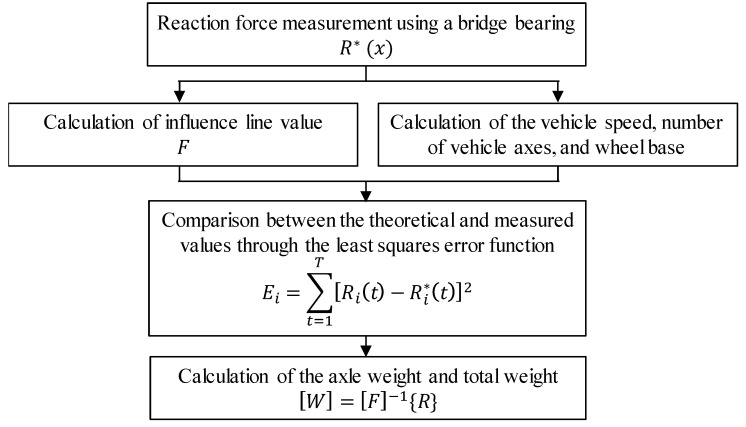
Method of estimating the vehicle load using reaction force response.

Figure 11 shows the method of estimating the vehicle load using the reaction force response. The influence line of a bridge and vehicle information are required to measure the vehicle load acting on the bridge. The information about the vehicle, including the number of vehicle axles, the wheel base, and vehicle speed, can be estimated using the reaction force response measured when the vehicle passes through the bridge. The vehicle load can be estimated when the error is the smallest in the theoretical reaction force response, derived by using the measured reaction force response and vehicle information. Therefore, it is possible to estimate the axial load and total load of the vehicle from the relationship between the measured response and influence line.

## 4. Vehicle Load Estimation Using Reaction Force Response in Numerical Analysis

### 4.1. Numerical Analysis Model

**Figure 12:**
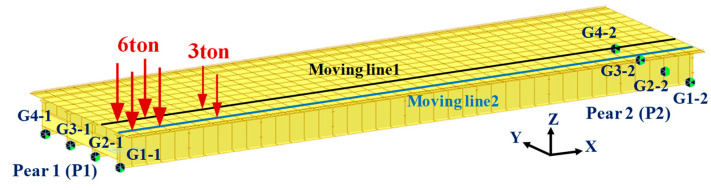
Numerical analysis model and the position of the vehicle load.

**Figure 13:**
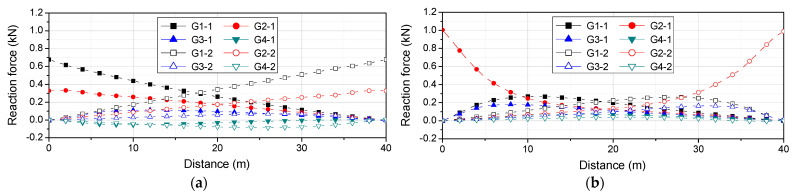
Influence line for the reaction force: (**a**) Moving line 1; (**b**) Moving line 2.

In this study, a short-span plate girder bridge was modeled, and a numerical analysis was conducted to examine the possibility of estimating the vehicle load using the reaction force response at the support. A numerical analysis was conducted using the MIDAS software [46]. In addition, the study examined the influence of noise and the delay of the polyurethane disk on the estimation of the vehicle load on a bridge. The plate girder bridge has a width of 12 m and a span of 40 m. As shown in Figure 12, the girder was modeled as a beam element and the slab as a shell element. A numerical analysis was conducted while the three-axial vehicle load was moved. When a vehicle passes through the bridge, the influence line for the reaction force can be expressed, as shown in Figure 13. An accurate influence line is essentially required because the vehicle load is estimated using the influence line for the reaction force and the reaction force response at the support.

### 4.2. Vehicle Load Estimation According to the Signal to Noise Ratio (SNR)

When the reaction force response is measured in the field, noise may occur due to impact and environmental factors. Consequently, changes in the axial load and total load of the vehicle according to SNR were examined to identify the relationship between the influence of noise and the estimated vehicle load. SNR can be expressed as Equation (21), where e(j) is a measured signal and s(j) is a signal without noise.

(21) $$\mathrm{SNR}=10\log_{10}\left(\overset{N}{\underset{j=1}{\sum }}s{\left(j\right)}^{2}/\overset{N}{\underset{j=1}{\sum }}{\left(e\left(j\right)-s\left(j\right)\right)}^{2}\right)\mathrm{dB}$$

**Figure 14:**
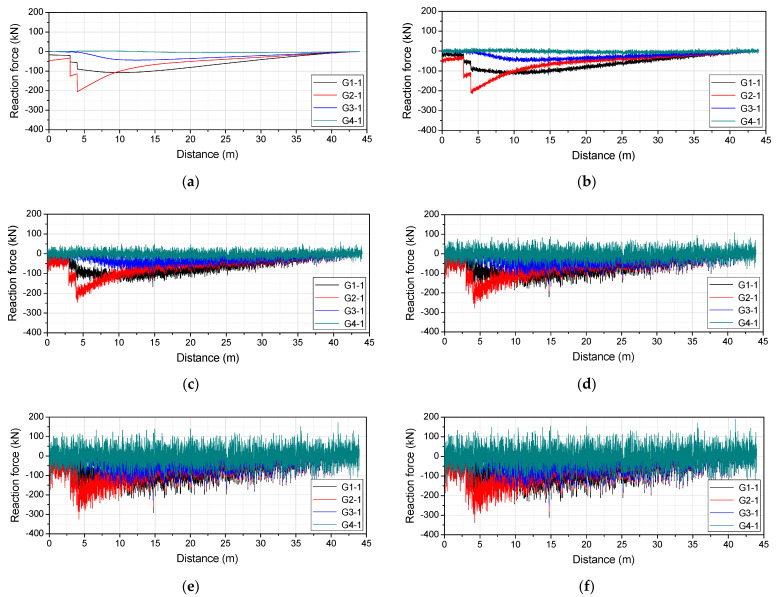
Reaction force response considering the influence of SNR: (**a**) response without noise; (**b**) SNR = 20 dB; (**c**) SNR = 10 dB; (**d**) SNR = 5 dB; € SNR = 1 dB; (**f**) SNR = 0.1 dB.

Figure 14a shows the results of the numerical analysis, i.e., the static reaction force response without noise depending on the position when the vehicle passes, as shown in Figure 12. In Figure 14a, the distance on the x-axis is the distance between the front axle of the vehicle and P1, which is point 1 in the pier. Figure 14b–f show the data created by adding white noise with all frequency domains to the reaction force response without noise. The error in the vehicle load, according to the magnitude of SNR was examined by varying the magnitude of SNR to 20, 10, 5, 1, and 0.1 dB, as shown in Table 5. As shown in the table, the error in the axial load and total load of the vehicle increased as the influence of noise increased. This indicates that the vehicle load with a small error can be estimated if the load is estimated using the measured response with a small influence of noise. At 0.1 dB, with the largest influence of noise, the error in the axial load was larger but that of the total load was less than 2%, confirming that the total load of a vehicle can be estimated despite the occurrence of noise.

### 4.3. Estimation of the Vehicle Load Considering the Delay of the Polyurethane Disk

The developed vertical displacement sensor measures the vertical load from the vertical displacement response of the polyurethane disk. Thus, an accurate vertical load measurement is only possible when the vertical displacement of the polyurethane disk is accurately calculated. Since the polyurethane disk has the properties of rubber, however, it involves a delay caused by a load. Therefore, the relationship between the delay and the estimated vehicle load was examined by estimating the vehicle load when the delay occurred. To consider the delay in this study, a data-smoothing method was applied to the data without noise. The application of the method caused data distortion at the peak of the reaction force response so that an effect similar to the delay could be considered. If the influence of this distortion on the response measured using the vertical displacement sensor developed can be identified, it will be possible to correct the vehicle load for the delay.

**Figure 15:**
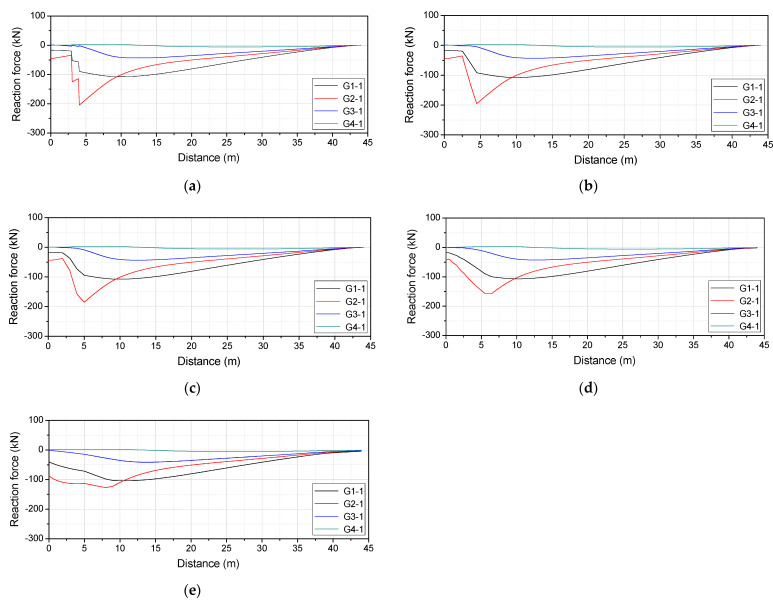
Reaction force response considering the delay: (**a**) N = 10; (**b**) N = 100; (**c**) N = 200; (**d**) N = 500; (**e**) N = 1000.

In this study, the simple moving average (SMA) technique was used to reduce the average of the overall error by dividing the data into small groups and then using their average values. SMA can be expressed as Equation (22), where $d_{i}$ refers to the measured response data and N is the number of small groups. Figure 15 shows the reaction force response when the number of small groups was changed to 10, 100, 200, 500, and 1000. The interval of the data is 0.01 m, and the intervals of the small groups are 0.1, 1, 2, 5, and 100 m.

(22) $$\mathrm{SMA}=\overset{N}{\underset{i=1}{\sum }}d_{i}/N$$

Table 6 shows the axial load and total load of the vehicle obtained using the reaction force response in Figure 15 that considered the delay. As shown in the table, errors in the axial load and total load increased as the number of small groups in SMA increased. Although the error in the axial load significantly increased as the delay increased, the error in the total load slightly increased. This shows that the total load can be estimated even if the estimation of the axial load is difficult when the delay is large.

When the vehicle load is estimated by considering the noise and delay of the reaction force response at the support, the numerical analysis will be more accurate when the difference between the theoretical response and the estimated response is smaller. This means that vehicle load estimation becomes more accurate as the mean deviation decreases between the theoretical response and the estimated response.

## 5. Vehicle Load Estimation Using the Vehicle Loading Test

### 5.1. Test Bridge

**Figure 16:**
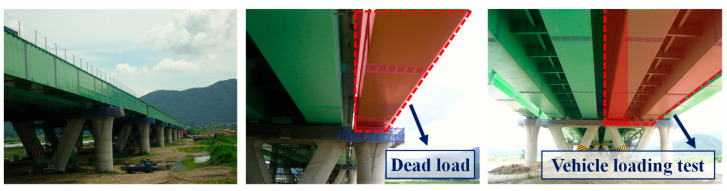
Test bridge.

To verify if it is possible for the developed FBG-based vertical displacement sensor to measure the dead load of a bridge and the load of a vehicle traveling on it, this study applied the sensor to the Yangsan Nakdong River Bridge located in Yangsan City, South Korea, as shown in Figure 16. The bridge on the Joongang Highway is a steel box girder bridge with a width of 24.210 m and a total length of 1,126.1 m, consisting of 16 spans and four lanes. The vehicle loading test was conducted in the 280 m (70@4 = 280 m) section of four spans and two lanes where the vertical displacement sensor was installed and access to a bridge bearing was convenient. The test bridge is a section intended to expand the existing bridge. The bridge bearing of the existing bridge was replaced with EQS, and the expansion part where EQS is installed was newly constructed and connected to the existing bridge.

### 5.2. Dead Load Estimation

**Figure 17:**
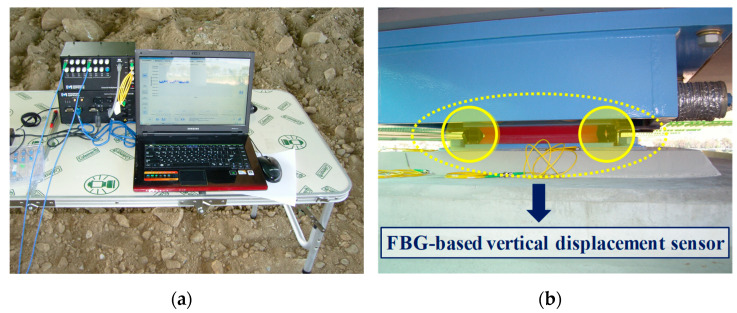
FBG measurement system and installed vertical displacement sensor: (**a**) FBG measurement system; (**b**) Installed vertical displacement sensor.

**Figure 18:**
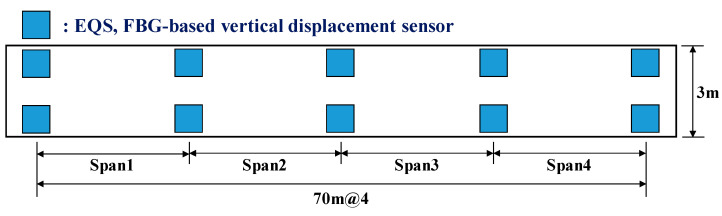
Vertical displacement sensor installation positions for dead load measurement.

Since only the steel box was installed in the expansion part, the dead load by the steel box was measured using the vertical displacement sensor. Figure 17a shows the system used to measure the FBG response, while Figure 17b shows the vertical displacement sensor applied to the bridge bearing with EQS. In measuring the reaction force response, one of the most important measurement factors in the field test as opposed to the laboratory test is the temperature. FBG is also used as a temperature sensor because it is temperature-sensitive. Therefore, in this study, the wavelength change due to temperature was measured by attaching an FBG sensor to the same material as the beam of the vertical displacement sensor next to the vertical displacement sensor, and thus the temperature error was corrected. Four vertical displacement sensors were installed at the edge of each bridge bearing to measure the reaction force caused by the dead load, as shown in Figure 18.

As shown in Table 7, the dead load for each span of the bridge was measured using the vertical displacement response acting on the support. When the measured load was compared with the design load, the load exhibited large errors in span but the total load showed only a small error. Since the bridge where the dead load was measured was under construction, the load distribution was not complete. This led to large errors in the dead load for each span. However, since the error for the total load was less than 0.05%, the usability of the developed vertical displacement sensor was found to be good. This means that dead load acting on a bridge can be measured using the vertical displacement sensor.

### 5.3. Vehicle Loading Test

**Figure 19:**
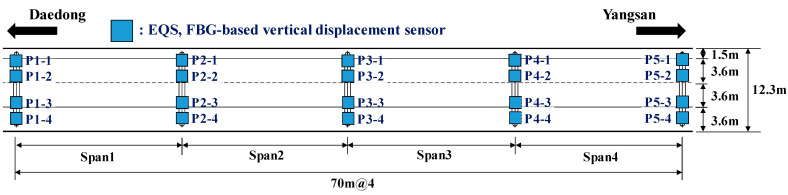
Vertical displacement sensor installation positions.

**Figure 20:**
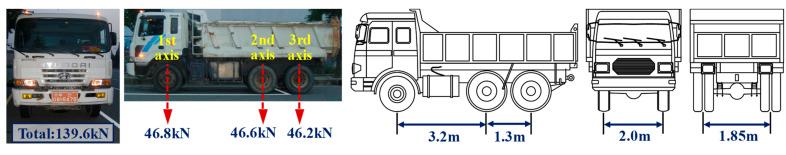
Specifications of the vehicle used in the vehicle loading test.

**Figure 21:**
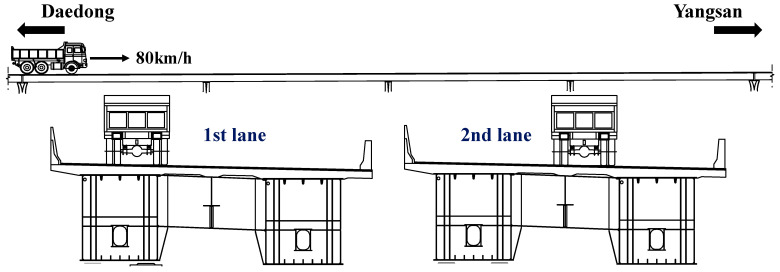
Direction and position of the vehicle traveling on the bridge.

The usability of the vertical displacement sensor developed for this study was examined by conducting a vehicle loading test of the bridge. The installation positions of the vertical displacement sensor are shown in Figure 19, and the data acquisition speed of the sensor was set to 125 Hz. Figure 20 shows the specifications of the vehicle used in the vehicle loading test. The total load of the vehicle was 139.6 kN. The vehicle loading test was conducted for cases in which the vehicle traveled on the first and second lanes of the bridge in the direction from Daedong to Yangsan at a speed of 80 km/h, as shown in Figure 21.

**Figure 22:**
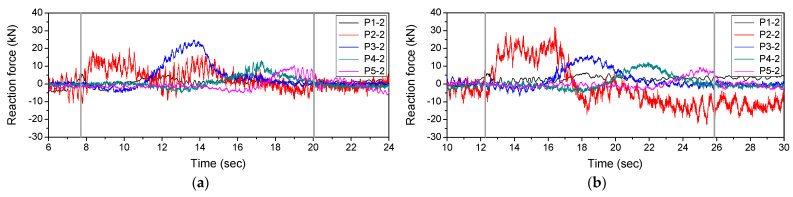
Reaction force response measured at the support of the bridge: (**a**) 1st lane; (**b**) 2nd lane.

The speed of the vehicle can be estimated from the difference between its bridge entry time and departure time and the length of the bridge. Figure 22 shows the reaction force response measured from P1-2 to P5-2, which is the support of the bridge, when the vehicle travelled on the first and second lanes. In Figure 22, the difference between entry time at P1-2 and departure time at P5-2 was measured. Table 8 shows the results of measuring the vehicle speed using Equation (8), which uses the difference between the bridge entry time and departure time and the length of the bridge. The driving speed of the vehicle is the speed confirmed on the driver’s seat instrument panel, and the estimated speed is the vehicle speed measured from the reaction force response. In Table 8, it was confirmed that the error between the estimated vehicle speed and the vehicle running speed was within 6%. The errors were attributed to the driver’s skills since the vehicle was not driven at a mechanically correct speed. Since the vehicle speed has a large effect on the result, the vehicle load was derived using the estimated vehicle speed.

**Figure 23:**
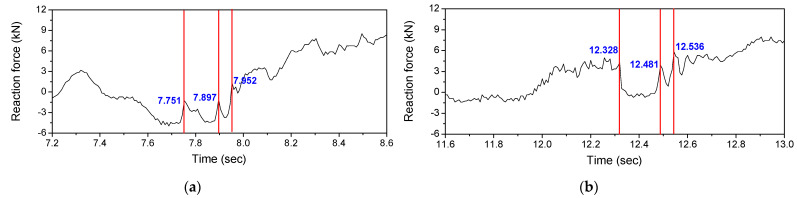
Reaction force response measured at P1: (**a**) 1st lane; (**b**) 2nd lane.

When the vehicle enters the bridge, an impact load is transmitted to the bridge due to the joint of the bridge. Therefore, as shown in Figure 23, the number of vehicle axles can be estimated from the number of impact loads measured at P1 where the vehicle enters the bridge. In addition, it is possible to measure the wheelbase of the vehicle from the time interval between the vehicle speed and the impact load. Table 9 shows the wheel base estimated from the estimated velocity of the vehicle and the time difference of the axes passing through one point. The difference between the estimated wheel base and the actual wheel base was less than 5%.

This difference occurred because the data acquisition speed of the vertical displacement sensor was 125 Hz and the time it took for the 4.5 m vehicle to pass one point at a traveling speed of 80 km/h was 0.2025 s. Since the time increment was 0.008 s, the distance traveled by the vehicle for 0.008 s was 0.18 m. Since the error of the wheel base is less than 0.18 m in Table 9, the error was found to be due to the size of the data acquisition speed. This indicates that the accurate wheel base of the vehicle can be measured if the data acquisition speed of the vertical displacement sensor is increased.

Since the load is estimated from the displacement response measured using the vertical displacement sensor in the test, a delay caused by polyurethane occurs when the load is being measured. Therefore, measuring the vehicle load using the influence line calculated in numerical analysis can differ from the influence line calculated through numerical analysis due to the material properties of the polyurethane disk. This indicates that accurate vehicle load estimation can be difficult if the influence line calculated through numerical analysis is used. Therefore, the influence line of the bridge was inversely estimated using the measured reaction force response. If the influence line for the reaction force at the support is used, the reaction force response at the support can be numerically expressed, as shown in Equation (23). Here, R(x) is the reaction force, $W_{n}$ is the n-th axial load, and r(x) is the influence line at vehicle position x on the bridge. It is possible to predict the influence line for the reaction force by substituting the accurate axial load of the vehicle traveling on the bridge and the measured reaction force response into Equation (24).

The influence line was predicted using the asymptotic method, which gradually finds the influence line from the difference between the arbitrarily entered initial influence line and the reaction force response. Equation (24) was obtained using Equation (23) to gradually predict the influence line. Here, $r_{0}\left(x\right)$ is the initial influence line and $r_{n}\left(x\right)$ is the estimated influence line. Equation (24) is a method of estimating the influence line by allowing it to gradually converge using the reaction force response and the initial influence line from the axial load of the vehicle.

(23) $$R\left(x\right)=W_{1}r\left(x\right)+W_{2}r\left(x-L_{1}\right)+W_{3}r\left(x-L_{2}\right)\;$$

(24) $$r_{1}\left(x\right)=r_{0}\left(x\right)+\frac{R\left(x\right)-\left\{W_{1}r_{0}\left(x\right)+W_{2}r_{0}\left(x-L_{1}\right)+W_{3}r_{0}\left(x-L_{2}\right)\right\}}{W}\;r_{2}\left(x\right)=r_{1}\left(x\right)+\frac{R\left(x\right)-\left\{W_{1}r_{1}\left(x\right)+W_{2}r_{1}\left(x-L_{1}\right)+W_{3}r_{1}\left(x-L_{2}\right)\right\}}{W}\;\vdots \;r_{n}\left(x\right)=r_{n-1}\left(x\right)+\frac{R\left(x\right)-\left\{W_{1}r_{n-1}\left(x\right)+W_{2}r_{n-1}\left(x-L_{1}\right)+W_{3}r_{n-1}\left(x-L_{2}\right)\right\}}{W}$$

**Figure 24:**
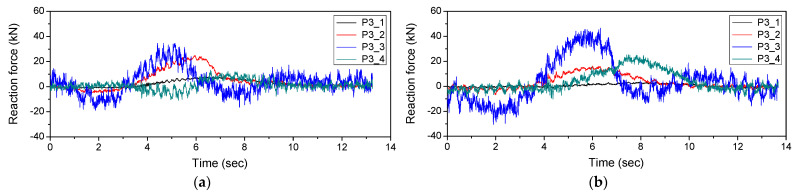
Reaction force response measured at P3: (**a**) 1st lane; (**b**) 2nd lane.

**Figure 25:**
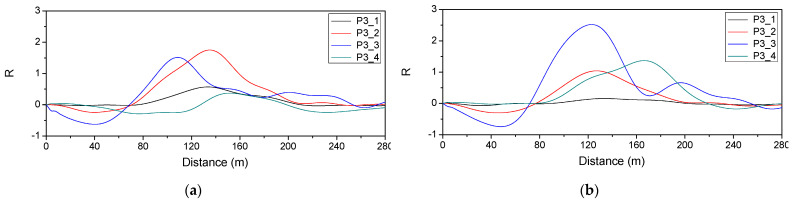
Estimated influence line: (**a**) 1st lane; (**b**) 2nd lane.

Figure 24 shows the reaction force response measured at P3 when the vehicle traveled on the first and second lanes. It is necessary to estimate the influence line depending on the lane traveled by the vehicle. Figure 25 shows the estimated influence line.

Table 10 shows the axial load and total load of the vehicle estimated using the influence line in Figure 25. It can be seen that the error rate of the total load is low but that of the axial load is high. Since the error rate for the total load was less than 5%, it was decided that estimating the total load is possible.

As the mean deviation between the theoretical and measured responses decreases, errors in the axial load and total load of the vehicle decrease. To reduce this mean deviation, data were filtered using a digital filter and the axial load and total load were subsequently estimated using the filtered data in this study. The measured reaction force response contains a large amount of noise from dynamic components that are not related to the natural frequency of the bridge. Therefore, a low-pass filter that only passes frequencies lower than a certain value and attenuates higher frequencies was applied to the digital filters.

**Figure 26:**
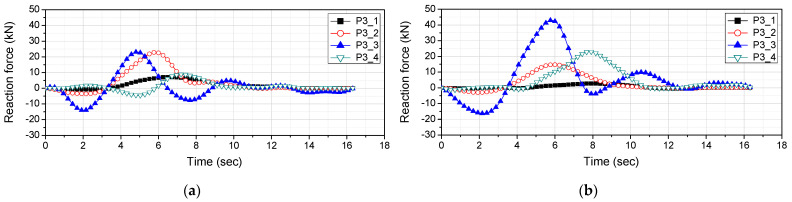
Reaction force response at P3 with the application of a low-pass filter: (**a**) 1st lane; (**b**) 2nd lane.

Figure 26 shows the reaction force response measured at P3 with the application of a low-pass filter. The axial load and total load were estimated using the reaction force response in Figure 26, as shown in Table 11. It was found that the application of a low-pass filter reduced errors in the axial load and total load. This indicates that the vehicle load can be more accurately estimated by reducing noise using a digital filter.

In Table 11, the axial load showed large errors, but the total load was relatively accurate. This is because the overall reaction force response was similar to the shape of the influence line. Therefore, estimation of the total load is deemed possible. Inaccurate estimation of the axial load was affected by the delay of the polyurethane disk. In addition, it was found that the impact loads generated at the joint of the bridge when the vehicle entered the bridge, and the large difference between the length of the bridge and the wheel base of the vehicle, caused large errors in the axial load.

## 6. Conclusions

This study proposed a method of measuring the vertical load by applying a vertical displacement sensor based on Fiber Bragg Grating (FBG) to the Eradi Quake System (EQS), a bridge bearing, to estimate the load of a vehicle traveling on a bridge. The developed vertical displacement sensor can estimate the load of a vehicle using the reaction force response at the support. EQS is equipped with a polyurethane disk that accommodates the reaction force response of the upper structure at all times. The reaction force response acting on EQS can be estimated by measuring the vertical displacement response of the polyurethane disk using the developed vertical displacement sensor.

It was found that, in compressive tests, the displacement response estimated using a depth micrometer and the vertical displacement sensor only contained small errors. This confirmed the accuracy of the developed vertical displacement sensor and verified the effectiveness of estimating the vertical displacement response using FBG. In the fatigue test, the vertical displacement sensor could continuously perform measurements, even after 200,000 cycles of fatigue loading, confirming its sufficient durability against fatigue.

The influence of noise and delay of the polyurethane disk on the estimation of the vehicle load on a bridge was examined using numerical analysis. The estimated axial load and total load of the vehicle showed larger errors as the noise and delay of the polyurethane disk increased. Despite the noise and delay of the polyurethane disk, however, estimation of the total load of the vehicle was possible. Therefore, this study confirmed that accurate vehicle load estimation is possible if the impact of noise and delay is reduced.

The vertical displacement sensor was applied to examine the possibility of measuring the dead load of a bridge and the load of a vehicle traveling on it. The dead load of a bridge under construction was measured using the vertical displacement sensor. Errors in dead load for each span were found to be large because the load distribution was incomplete. As the difference in total load was not significant, however, the reliability of the vertical displacement sensor was found to be high. The applicability of the sensor was verified by conducting the vehicle loading test on a bridge. The test results showed that it is possible to estimate the number of vehicle axles and wheel base and the vehicle’s total load. It was also found that the vehicle’s total load could be estimated with only a small error when the mean deviation was reduced by applying a digital filter to the measured response. It was confirmed, however, that the large difference between the wheel base of the vehicle and the total length of the bridge, as well as the large influence of the delay of the polyurethane disk, resulted in large errors in axial load. Since the delay of the polyurethane disk causes large errors in the axial load, further study is required to reduce errors in the axial load from the correlation between the vehicle speed and the delay.

Various types of bridge bearings have been applied to bridges and, in this study, an FBG-based vertical displacement sensor that can be applied to EQS was developed. Application of the vertical displacement sensor to various other bridge bearings, including pot bearings and elastic bearings, can be checked by further study applying FBG to various types of bridges. When the number of vehicles that pass through a bridge is very high, however, it is difficult to determine the type and load of the vehicle using only the reaction force response of the bridge support. Therefore, additional studies need to be conducted to measure the types and loads of vehicles that are constantly traveling on the bridge, in conjunction with video information to check the actual vehicle traffic transpiring in that bridge. In this way, it will be possible to identify the fatigue damage on the bridge structure due to the actual load and characteristics of its vehicle traffic.

## Figures and Tables

**Table 1:** Specification of FBG-based vertical displacement sensor.

Size		Beam		FBG	
width	45 mm	material	stainless Steel	centre wavelength tolerance	± 0.5 nm
				peak reflectivity	>= 70%
depth	40 mm	width	13 mm	FBG length	5 mm
				fiber original coating	polyimide
		thickness	0.1 mm	fiber re-coating type	polyimide
height	57 mm	height	53 mm	fiber type	SMF28-C

**Table 2:** Forced displacement of the depth micrometer and displacement measurements by vertical displacement sensors.

Forced Displacement (mm)	Measured Displacement (mm), Error Rate (%)			
	Sensor 1	Sensor 2	Sensor 3	Sensor 4
1	0.996 (0.400)	1.004 (0.400)	1.013 (1.300)	0.982 (1.800)
2	1.987 (0.650)	2.003 (0.150)	2.025 (1.250)	1.977 (1.150)
4	3.982 (0.450)	4.007 (0.175)	4.054 (1.350)	3.979 (0.525)
6	5.963 (0.617)	6.011 (0.183)	5.957 (0.717)	6.019 (0.317)
8	7.953 (0.587)	8.015 (0.188)	7.968 (0.400)	8.074 (0.925)
10	9.955 (0.450)	9.993 (0.070)	10.025 (0.250)	10.042 (0.420)

**Table 3:** Error analysis for the displacement response measured using the vertical displacement sensor.

Case	Percent Error [%]	RMS Error [mm]
1	0.065	0.116
2	0.173	0.184

**Table 4:** Displacement measured using the vertical displacement sensor according to the number of load cycles.

Number of Load Cycles (Cycle)	Minimum Displacement (mm)	Maximum Displacement (mm)	Difference (mm)
50,000	5.364	5.626	0.262
100,000	5.412	5.674	0.262
150,000	5.423	5.685	0.262
200,000	5.432	5.694	0.262

**Table 5:** Comparison of the vehicle loads, estimated considering the influence of SNR.

SNR	Vehicle Load (kN), Error Rate (%)			
	1st Axis	2nd Axis	3rd Axis	Total
without noise	60.000	120.000	120.000	300.000
20 dB	60.540 (0.900)	119.903 (0.081)	120.060 (0.050)	300.503 (0.168)
10 dB	61.708 (2.847)	119.693 (0.256)	120.189 (0.157)	301.590 (0.530)
5 dB	63.038 (5.062)	119.454 (0.455)	120.336 (0.280)	302.827 (0.942)
1 dB	64.815 (8.024)	119.134 (0.721)	120.532 (0.443)	304.481 (1.494)
0.1 dB	65.340 (8.900)	119.040 (0.800)	120.590 (0.492)	304.970 (1.657)

**Table 6:** Comparison of vehicle loads considering the delay.

N	Vehicle Load (kN), Error Rate (%)			
	1st Axis	2nd Axis	3rd Axis	Total
10	60.391 (0.651)	119.468 (0.444)	120.116 (0.097)	299.975 (0.008)
100	63.694 (6.156)	114.916 (4.237)	121.116 (0.930)	299.725 (0.092)
200	67.522 (12.537)	109.533 (8.723)	122.306 (1.922)	299.360 (0.213)
500	89.182 (48.636)	78.975 (34.187)	129.364 (7.804)	297.521 (0.826)
1000	151.677 (152.794)	16.094 (86.588)	126.767 (5.639)	294.537 (1.821)

**Table 7:** Dead load for each span of the bridge.

Dead Load	Span1	Span2	Span3	Span4	Total
design load (kN)	1687.812	1190.114	1360.857	1845.538	6084.293
measured load (kN)	1579.814	1282.878	1451.636	1766.887	6081.132
difference (kN)	107.998	−92.764	−90.779	78.651	3.161
error rate (%)	6.40	7.79	6.67	4.26	0.05

**Table 8:** Vehicle speed estimated from the reaction force response.

Lane	Travel Velocity (km/h)	Estimated Velocity (km/h)	Error Rate (%)
1	80	82.751	3.439
2	80	75.619	5.475

**Table 9:** Estimated wheel base of the vehicle.

Lane	Wheelbase (m), Error Rate (%)		
	1st~2nd Axis (3.2 m)	2nd~3rd Axis (1.3 m)	1st~3rd Axis (4.5 m)
1	3.337 (4.281)	1.348 (3.692)	4.687 (4.133)
2	3.209 (0.281)	1.272 (2.154)	4.578 (1.733)

**Table 10:** Vehicle load measurement using the vertical displacement sensor developed.

Lane	Vehicle Load (kN), Error Rate (%)			
	1st Axis (46.8 kN)	2nd Axis (46.6 kN)	3rd Axis (46.2 kN)	Total (139.6 kN)
1	89.763 (91.801)	−83.892 (280.026)	134.229 (190.539)	140.100 (0.358)
2	−303.219 (747.904)	1195.781 (2466.054)	−757.929 (1740.540)	134.633 (3.558)

**Table 11:** Vehicle load with the application of the low pass filter.

Lane	Vehicle Load (kN), Error Rate (%)			
	1st Axis (46.8 kN)	2nd Axis (46.6 kN)	3rd Axis (46.2 kN)	Total (139.6 kN)
1	46.902 (0.218)	33.117 (28.933)	59.580 (28.961)	139.599 (0.001)
2	46.831 (0.066)	34.425 (26.127)	58.342 (26.281)	139.599 (0.001)

## Data Availability

Not applicable.
